# The critical roles of COUP-TFII in tumor progression and metastasis

**DOI:** 10.1186/2045-3701-4-58

**Published:** 2014-10-01

**Authors:** Jun Qin, Sophia Y Tsai, Ming-Jer Tsai

**Affiliations:** Department of Urology, Renji Hospital Shanghai Jiao Tong University School of Medicine, Shanghai, 200127 China; Institute of Health Sciences, Shanghai Institutes for Biological Sciences, Chinese Academy of Sciences, Shanghai, People’s Republic of China; Shanghai JiaoTong University School of Medicine, Shanghai, People’s Republic of China; Department of Molecular and Cellular Biology, Baylor College of Medicine, One Baylor Plaza, Houston, TX 77030 USA; Program in Developmental Biology, Baylor College of Medicine, Houston, TX USA

## Abstract

Chicken ovalbumin upstream promoter transcription factor II (COUP-TFII) belongs to the steroid/thyroid hormone receptor superfamily. Extensive evidence has indicated that COUP-TFII plays a critical and indispensable role in cell-fate specification, organogenesis, angiogenesis, and metabolism as well as in a variety of diseases. Recent studies obtained from genetically engineered mouse models (GEM) and patient specimen analysis indicate that COUP-TFII is also important for tumor progression and metastasis. In this article, we will comprehensively review the oncogenic roles of COUP-TFII within the tumor microenvironment and tumor cells and delineate the mechanism by which COUP-TFII contributes to tumorigenesis. The applicability of current data to our understanding of the role of COUP-TFII in cancer and the potential therapeutic implications will also be discussed.

The nuclear receptor (NR) superfamily of ligand-activated receptors exhibit a common modular structure and play essential roles, not only in maintaining cellular homeostasis, but also in various disease processes including cancer and metabolism disorder [1, 2]. Chicken ovalbumin upstream promoter transcription factor II (COUP-TFII, also named as NR2F2), a member of the steroid/thyroid hormone receptor superfamily, was originally identified to be a transcriptional factor regulating the expression of the chicken ovalbumin gene in chicken oviducts [3]. COUP-TFII possesses the classic domain structure of nuclear receptors. Specifically, it encompasses two highly conserved motifs: 1) a DNA-binding domain (DBD) containing two zinc-fingers; and 2) a putative ligand-binding domain (LBD) (Figure 1A) [4]. COUP-TFII can activate or repress gene expression in both a tissue-specific and gene-specific manner through mechanisms involving direct binding to DNA response elements or binding to other transcription factors. Through binding to 5’-AGGTCA-3’ direct repeats (DR) with variable spacing, COUP-TFII represses gene expression through the recruitment of CoR (corepressor) (Figure 1B) [4]. Alternatively, COUP-TFII can also bind to Sp1 sites to cooperatively activate gene expression such as Angiopietin-1 (Ang-1) and Neuropilin 2 (Nrp2) (Figure 1B) [5, 6].

**Figure 1:**
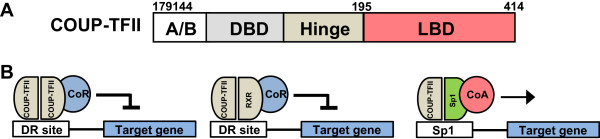
**Schematic structure COUP-TFII and known mechanism of its transcriptional regulation. A)** Schematic structure of human COUP-TFII proteins. DNA-binding domain (DBD); ligand-binding domain (LBD) **B)** COUP-TFII binds to 5′-AGGTCA-3′ motif palindromes (DR site), either directly (homodimer) or indirectly, through heterodimer formation with other proteins (e.g. RXRs) to regulate downstream target gene expression. In addition, COUP-TFII can also bind to Sp1 sites through interaction with Sp1 to cooperatively activate gene expression.

In the past decades, great insights have been obtained into the physiological function of COUP-TFII during embryonic and postnatal development. Using genetically engineered mouse models (GEM) together with molecular analysis, it has been well documented that COUP-TFII serves as one of the master regulators to control developmental programs, including organogenesis, angiogenesis, cardiovascular development, reproduction, neuronal development and metabolic homeostasis [7–15]. Mechanistic investigations uncover that COUP-TFII exerts its function through modulation of cell proliferation, migration, survival, fate determination and differentiation in a context dependent manner. Aside from its critical roles in physiological process [10], recent studies also reveal that COUP-TFII plays important roles in pathological processes such as cancer [5, 6, 16–19], congenital diaphragmatic hernia (CDH) [12] and diabetes [11]. Since the function of COUP-TFII in developmental processes has been extensively reviewed recently [20], here we will focus on its role in tumorigenesis as well as its underlying mechanism, and discuss potential therapeutic implications for cancer intervention.

## Role of COUP-TFII in the tumor microenvironment

**Figure 2:**
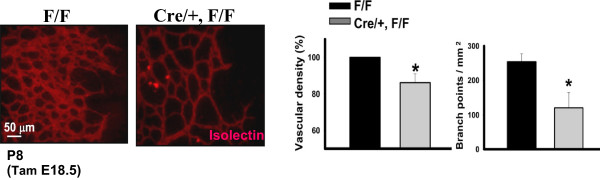
**Roles of COUP-TFII in retinal vascular development.** Analysis of changes in retinal microvasculature by Isolectin B4 staining of retinal vessels from P8 mice. Animals were treated with tamoxifen at E18.5 to induce COUP-TFII deletion at the postnatal stage. Relative Isolectin B4-positive density and number of vessel branch points are graphed as mean (±SEM) (N = 10) * P < 0.05. We showed that the complexity and density of the vascular network were substantially reduced in the mutant retina in comparison with the control retina. F/F: COUP-TFII^Flox/Flox^; Cre/+, F/F: Rosa26^CreERT2/+^, COUP-TFII^Flox/Flox^.

COUP-TFII is highly expressed in the mesenchymal cell compartment during embryogenesis, whereas its expression is relatively low in the adult epithelium [10]. Thus, it is not surprising that ablation of COUP-TFII in adults lacks a discernible phenotype [6, 21]. In most instances, COUP-TFII is not important for maintenance function, but it is essential for regeneration or dedifferentiation processes, which often occur under pathological conditions in adults. Given the fact that disease malignance often shares similar mechanisms as developmental processes, the knowledge gained from studying the COUP-TFII knockout mice in the past has a major impact on the understanding of the disease processes. At embryonic day 9.5 (E9.5), COUP-TFII null mutants display a severe defect in angiogenesis as evidenced by the observations that the primitive capillary plexus fails to undergo remodeling to generate large and small microcapillaries as well as the inability of the capillary to invade into areas lacking blood vessels [15]. Likewise, depletion of COUP-TFII in the postnatal stage significantly compromised blood vessel formation by using retina angiogenesis as a neo-angiogenesis model (Figure 2). As we all know, cancer cells often hijack a variety of normal cellular processes to enable survival and expansion in an organism, and the above observations raise an intriguing possibility that COUP-TFII might be critical for tumor angiogenesis, which often shares similar genetic pathways with neo-angiogenesis. In agreement with this notion, our lab has identified that COUP-TFII serves as one of the major angiogenic regulators within the tumor microenvironment to promote tumor angiogenesis in a spontaneous breast cancer model (MMTV-PyMT) and pancreatic cancer model (RIP1-Tag2) [6, 10, 18, 19].

It is well accepted that tumor progression, invasion, and metastasis requires blood vessels and lymphatic vessels to provide oxygen and nutrients to sustain tumor growth [21]. In COUP-TFII mutant mice, vessel growth is dampened, rendering tumor cells lacking blood supply and undergoing apoptosis or necrosis [6, 19]. As a consequence, tumor progression is inhibited in COUP-TFII depleted mice. More importantly, impeding the paths for tumors to escape from the primary sites into the circulating system, tumor metastasis is also inhibited with the loss of COUP-TFII. Further mechanistic investigations uncover that COUP-TFII modulates multiple angiogenic signals (VEGF/VEGFR-2, Angpoietin/Tie2, Notch and E2F-1 growth signaling) to regulate tumor angiogenesis [6, 18, 19]. In pericytes, COUP-TFII directly regulates Ang-1 expression. Ang-1/Tie2 signaling serves as a paracrine signal for vascular remodeling and maturation. In addition, COUP-TFII plays a cell autonomous role within endothelial cells to promote vessel sprouting. Depletion of COUP-TFII significantly inhibits endothelial cell sprouting partly through transcriptional derepression of vascular endothelial growth factor receptor 1 (VEGFR-1). Given the fact that the soluble and the membrane bound VEGFR-1 potently sequester VEGF binding to the vascular endothelial growth factor receptor 2 (VEGFR-2), VEGF/VEGR-2 signaling [21], a major driving force for tumor angiogenesis, is inhibited upon COUP-TFII depletion. The functional relevance between COUP-TFII and VEGFR-2 signaling is further substantiated by rescue experiments in which neutralization of VEGFR-1 overexpression caused by COUP-TFII ablation partially restores vessel sprouting concomitant with the hyperactivation of VEGF/VEGR-2 signaling. Moreover, we also demonstrated that COUP-TFII directly controls E2F1 expression and represses Notch signaling in endothelial cells to modulate cell proliferation and migration [18]. Taken together, the above results indicate that COUP-TFII directly regulates the transcription of three key genes, E2F1 and VEGFR-1 in endothelial cells, and Ang-1 in pericytes, to coordinate endothelial cell proliferation, sprouting, and vascular remodeling [6, 18, 19]. Interestingly, COUP-TFII is expressed not only in the venous endothelial cells [12], but also in the lymphatic endothelial cells (LECs) [5]. Lin et al. identified that COUP-TFII controls sprouting lymphangiogenesis through direct transcriptional regulation of Nrp2, a co-receptor for VEGF-C and thereby modulates the VEGF-C/VEGFR-3 signaling pathway in the lymphatic system [5]. As expected, inactivation of COUP-TFII in the tumor microenvironment substantially suppresses tumor-induced neo-lymphangiogenesis. Given the facts that there is limited success for anti-angiogenic therapy (Bevacizumab and Sunitinib) in patients and COUP-TFII potently targets multiple angiogenic and lymphangiogenic pathways within the tumor microenvironment, these studies prompted the rational basis that COUP-TFII might represent a potential therapeutic target and inhibition of COUP-TFII may offer an efficacious approach for anti-angiogenic intervention.

**Figure 3:**
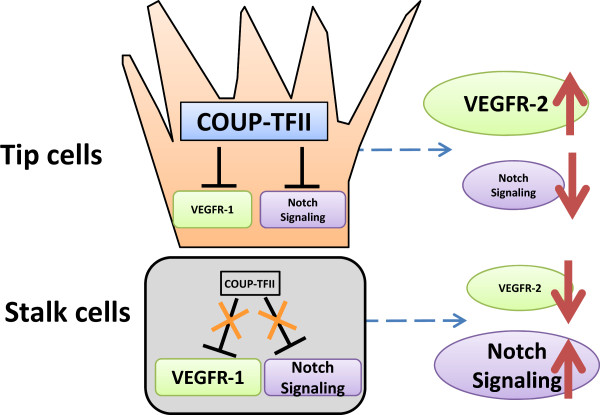
**Potential model for COUP-TFII function in tip and stalk cell specification during angiogenesis.** COUP-TFII is preferably expressed in tip cells to ensure the inactivation of Notch signaling and activation of VEGFR-2 signaling during the sprout process. In stalk cells, Notch signaling and VEGFR-1 expression will be up-regulated due to the absence of COUP-TFII.

One important question that remains to be defined is whether COUP-TFII is involved in tip and stalk cell specification during vessel sprouting. During angiogenesis, reciprocal inhibition between Notch and VEGFR-2 signaling has been shown to be critical for the tip and stalk cell specification [22–24]. We have shown that COUP-TFII activates VEGFR-2 signaling *via* direct repression of VEGFR-1 expression [19]. In addition, COUP-TFII antagonizes Notch signaling through direct regulation of players at multiple steps of the Notch cascade including Foxc1, Np-1, and Hey2 [18]. Thus, it is reasonable to speculate that COUP-TFII is preferably expressed in tip cells to ensure the inactivation of Notch signaling and activation of VEGFR-2 signaling during the sprouting process (Figure 3). On the other hand, Notch signaling and VEGFR-1 expression will be expected to be up-regulated in stalk cells due to the absence of COUP-TFII. Thus, these complicated hierarchies of signaling interactions are coordinated, and the orchestration among COUP-TFII, VEGF/VEGFR-2 signaling, and Notch signaling remains to be further elucidated, which might provide new insights for the underling mechanism of tumor angiogenesis and identify new targets for anti-angiogenic therapy.

## The cell autonomous role of COUP-TFII in tumor cells

### COUP-TFII in prostate cancer

In contrast to the expression profile observed in normal tissue, aberrant COUP-TFII expression has been frequently reported in different types of human tumors including breast cancer [25, 26], pancreatic adenocarcinoma [27], colorectal cancer [28], ovarian cancer [29–31] and prostate carcinomas [16]. Therefore, the important question is whether COUP-TFII also plays an important role in tumor cells to modulate tumor progression and metastasis. To address this issue, COUP-TFII expression in more than 400 prostate cancer patient specimens has been evaluated. The results indicate that the expression of COUP-TFII is significantly elevated in prostate tumors, and further increased in metastatic prostate cancer patients [16]. Approximately 60% of all tumors exhibit intermediate to intense staining, whereas only 5% of the non-tumor tissue stained positive for COUP-TFII. More importantly, COUP-TFII expression in prostate tumor cells correlates with increased risk of tumor recurrence and decreased survival after prostatectomy, suggesting that COUP-TFII is a potential biomarker for aggressive PCa diagnosis. Furthermore, the results that COUP-TFII-deficiency in association with PTEN loss profoundly impacts prostate tumorigenesis, affecting the size, progression, and severity of lesions, indicating that COUP-TFII plays a causal role in disease progression. More importantly, prostate-specific overexpression of COUP-TFII cooperates with PTEN loss to produce an invasive cancer and promotes the metastasis of tumor cells to distant tissues. Taken together, these results indicate an oncogenic role of COUP-TFII in driving the indolent tumor to become a metastatic-prone prostate cancer.

**Figure 4:**
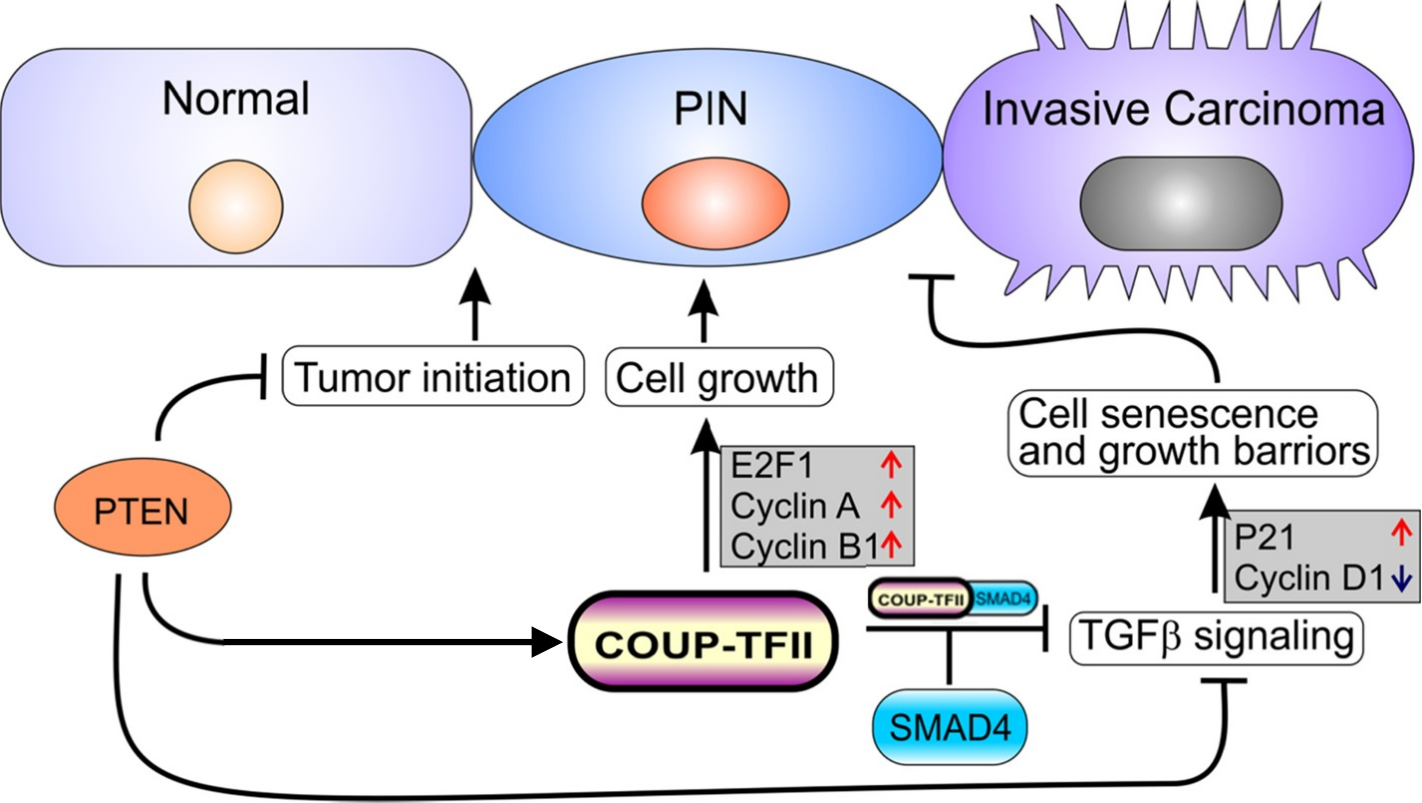
**Working model for COUP-TFII function in prostate tumorigenesis.** PTEN inactivation results in an increase of COUP-TFII expression in prostate cancer cells, which will consequently promote tumor growth through the regulation of cell cycle or growth related genes such as E2F1, Cyclin A, Cyclin B and etc. In addition, further increase in COUP-TFII expression in prostate cancer constrains the TGF-β-dependent growth barrier through direct association with Smad4, thereby downregulating p21 expression and enhancing Cyclin D1 expression. In summary, COUP-TFII serves as an important regulator to control PTEN-mediated prostate tumorigenesis and renders the indolent tumors to acquire metastatic potential through destruction of the TGF-β-dependent growth barrier.

Mechanistic investigations further reveal that COUP-TFII associates with Smad4 and inhibits its transcriptional activity, and consequently counteracts the TGF-β signaling induced growth barrier for PTEN null indolent tumors [16]. The functional antagonism between COUP-TFII and Smad4 is further evidenced by GEM, in which conditional ablation of Smad4 rescues the invasive tumor growth in mice lacking COUP-TFII, and overexpression of COUP-TFII does not exacerbate tumor malignance further in the absence of Smad4. Multivariate analysis in patients further substantiates the functional relevance of COUP-TFII and TGF-β signaling in prostate cancer. The COUP-TFII signature carries an independent predictive value to further enhance the prognostic accuracy of four genes’ prediction, including PTEN, Smad4, p21 and CyclinD1. Taken together, these results demonstrate that COUP-TFII-mediated inhibition of TGF-β signaling is important for the PTEN-null prostate to develop into fully penetrated prostate cancer (Figure 4).

Having established that COUP-TFII serves as a critical regulator to counteract the TGF-β-induced growth barrier, the detailed mechanism remains to be further dissected. These phenotypes rely in part on inhibition of TGF-β signaling via physical interaction with Smad4. As a consequence, the downstream target genes of TGF-β relevant to growth response, such as p21, p15 and Cyclin D1, are deregulated, which will lead to cell senescence and inhibition of cell growth in COUP-TFII deficient mice (Figure 4). COUP-TFII sequesters Smad4 binding to target genes, which will be one of the mechanisms for COUP-TFII inhibitory effects on TGF-β signaling. However, as a transcription factor, COUP-TFII might also recruit co-repressors to the complex or disrupt the bindings of Smads to the co-activators such as CBP/P300. In addition to TGF-β signaling, COUP-TFII also induced a specific set of genes for cell growth and metastasis to fully establish malignant phenotypes. Analyses of gene expression arrays indicate that many cell growth and metastasis related genes, including E2F1, Cyclin B, SRC-3, FoxO1, FoxM1 and MMPs, are dysregulated upon COUP-TFII depletion. We have identified that COUP-TFII can directly control E2F1 transcription in a Sp1 dependent manner. E2F1 is known to be a major regulator for G1/S phase transition [32]. Thus, the mechanism that COUP-TFII promotes tumor progression and metastasis is not limited to its modulation of anti-proliferative effects of TGF-β signaling.

TGF-β has biphasic effects during tumorigenesis, acting early as a tumor suppressor, but later stimulating cancer progression through its effect on the tumor cells and their microenvironment [33]. It seems elusive that overexpression of COUP-TFII promotes cancer metastasis and at the same time inhibits TGF-β signaling. However, we believe that tumors bearing high levels of COUP-TFII will be resistant to growth inhibitory effects caused by TGF-β signaling and will enable the tumor cells to accumulate mutations and gain metastatic potential. Since TGF-β signaling is hypoactivated upon COUP-TFII overexpression, COUP-TFII might modulate other pathways or regulators to promote tumor metastasis, which has yet to be further elucidated.

### COUP-TFII in other types of cancer

It is reasonable to speculate that COUP-TFII might be involved in other cancer types in addition to prostate cancer. Indeed, COUP-TFII is among a limited group of NRs that are prognostic for breast cancer classification and histologic grade. In 119 human breast cancer patients, fifty-nine percent of the specimens are immunohistochemically positive for COUP-TFII, and COUP-TFII expression level positively correlates with a poor clinical stage, lymph node status, histological grade and estrogen receptor alpha status [25]. However, in contrast to this finding, Litchfield et al. reported that COUP-TFII expression is negatively associated with clinical outcome and disease progression [26]. Though COUP-TFII expression is higher in ERα + breast cancer samples, its expression is significantly lower in metastatic samples [26]. In addition, COUP-TFII is reduced in tamoxifen-resistant human breast cancer cells, and ectopic expression of COUP-TFII renders the cells to be tamoxifen sensitive [34]. Thus, the prognostic significance of COUP-TFII varies between studies for breast cancer, and whether this is due to different subsets of patients needs to be clarified.

In ovarian cancer, it is generally believed that COUP-TFII expression is downregulated in comparison with normal counterparts [29–31]. However, one needs to take into account that COUP-TFII is highly expressed in the stroma of a healthy ovary with little or no expression in the epithelium. Thus, downregulation of COUP-TFII in ovarian cancer based on microarray data may only reflect the composition changes between tumor and normal tissue, since tissue compartmental ratios (epithelial versus stromal) have been significantly altered once a tumor is formed. Using tissue microarray assay, recent studies partially resolved this puzzle [30]. Interestingly, they found that ectopic epithelial expression of COUP-TFII is frequently observed in ovarian cancer patients. The frequency of COUP-TFII staining in the epithelium of metastatic ovarian cancers is significantly higher than the levels observed in the indolent ones, and tumors having higher COUP-TFII expression in the epithelium is associated with a trend toward greater likelihood of disease recurrence [30], supporting an oncogenic role of COUP-TFII in ovarian cancer at least in the epithelium compartment. In pancreatic adenocarcinoma, COUP-TFII is expressed in 69% of tested primary samples and correlates with the N1 and M1 status and clinical stage; Kaplan-Meier and Cox regression analysis show that it may be an independent prognostic factor of a worse outcome [27]. In vitro silencing of COUP-TFII reduces the cell growth and invasiveness. In colon cancer, COUP-TFII is observed in more than 57% of tumors from colon cancer patients, and there was minimal expression in normal colonic mucosa; expression of the receptor correlated with increased rates of disease-free survival. Taken together, the prognostic significance of COUP-TFII varies between studies and cancer types, and the precise roles of COUP-TFII in cancer progression and metastasis are still elusive. These differences reported in literature may be due to the intrinsic differences among various cancer types and COUP-TFII may exert its functions in a context dependent manner.

## Concluding remarks

This review has highlighted a critical role of transcription factor COUP-TFII in tumor progression and metastasis. In prostate tumor cells, COUP-TFII counteracts the TGF-β-dependent growth barrier to drive prostate tumor growth and metastasis (Figure 4) [16]. In addition, COUP-TFII in the tumor microenvironment promotes angiogenesis to facilitate growth and metastasis of breast and pancreatic tumors [6, 18, 19]. Since nuclear receptors, whose activity can be regulated by small molecular compounds [1], are ideal targets for drug discovery [35], COUP-TFII might represent as an excellent and “druggable” target for cancer intervention. Indeed, X-ray crystallography indicates that COUP-TFII contains a ligand-binding pocket whose activity can be regulated by small diffusible ligands [36]. In accordance with this notion, 9-cis- and all-trans retinoic acid induces COUP-TFII-dependent transactivation and enhances coactivator binding [36]. These findings encourage us to screen and identify the specific COUP-TFII antagonists or inhibitors and examine its effects in pre-clinical cancer models, which can be potentially used for cancer intervention.

Although tremendous progress have been made regarding to the roles of COUP-TFII in tumorigenesis, there are many questions that remain to be addressed. Given the fact that COUP-TFII is generally important for cellular differentiation and lineage determination, it leaves open the possibility that COUP-TFII might be involved in cancer stem cells homeostasis. Specifically, it is interesting to address whether COUP-TFII is involved in the re-programming of terminally differentiated cells into dedifferentiated cancer stem cells or tumor progenitor cells. Also, what is role of COUP-TFII in drug resistance? For instance, since COUP-TFII was reported to interact with androgen receptor (AR) to modulate its transcription activity in prostate cancer cells [37], COUP-TFII might be involved in the development of castration resistance prostate cancer (CRPC). Finally, having established the oncogenic role of COUP-TFII in prostate cancer, we need to address the precise roles of COUP-TFII in other cancer types. Based on patient data mentioned above, we believe COUP-TFII exerts its function in a context and cancer cell type dependent manner.

Except for the pathological roles of COUP-TFII, the detailed mechanism for COUP-TFII action remains to be further elucidated. First, what is the underlying mechanism for COUP-TFII driving tumor cells to become more metastatic? COUP-TFII might be involved in epithelial-mesenchymal transition to facilitate tumor cells metastasis and subsequently colonization in the distant organs. As a transcription factor, it is unclear how COUP-TFII activates the expression of some target genes, while at the same time represses the expression of other sets of genes. Further identification of the COUP-TFII interactome and transcriptome will help to address this complicated issue. In addition, as a nuclear protein, how does COUP-TFI respond to extracellular insults to coordinate its function? For instance,which oncogenic insults are responsible for the upregulation of COUP-TFII expression in prostate cancer cells? Addressing the questions raised above will help us understand further the signaling crosstalk mediated by COUP-TFII during tumorigenesis. In summary, future studies, including genome-wide approaches and detailed analysis of COUP-TFII-dependent genetic programs will be essential to comprehensively understand the roles of COUP-TFII in tumor progression and metastasis.
